# Editorial: Adapted & (dis)ability sport

**DOI:** 10.3389/fpsyg.2024.1515330

**Published:** 2024-11-19

**Authors:** Derek M. Peters, Rune Høigaard, John William Francis

**Affiliations:** ^1^University of Worcester, Worcester, United Kingdom; ^2^Department of Sport Science and Physical Education, University of Agder, Kristiansand, Norway

**Keywords:** disability, sport, adapted sport, psychology, performance analysis, physiology, socio-cultural

In recent years, international policies and priorities have increasingly aligned to raise awareness of adapted and (dis)ability sports (Hammond et al., 2022). This alignment has significantly elevated the profile and presence of these sports at all levels, leading to greater public, private, and media engagement globally. As a result, there are now more opportunities than ever for participation, viewing, and research in the field. With this heightened societal awareness and support, it is crucial to centralize, promote and enhance the status of high-quality research and knowledge exchange across all academic disciplines. This will deepen our understanding of every aspect of adapted and (dis)ability sports.

However, despite these advances, current research in this area is often fragmented, published in single-discipline-focused books or collections, and frequently concentrating on one sport, specific adaptions or (dis)ability, or a primary focus on the Paralympic movement (Kohe and Peters, 2017). While this research is valuable, its placement in broader non-(dis)ability-focused sports publications limits its accessibility and impact. As the field continues to grow, it is essential to foster interdisciplinary collaborations that incorporate various perspectives, physiological, psychological, technical, tactical and sociological, to fully understand and improve the experiences, wellbeing and performances of athletes with (dis)abilities.

In response to these challenges, this Research Topic was initiated to address this by creating a critical mass of research, knowledge exchange and contributions from authors representing all disciplines relevant to the study of adapted and (dis)ability sports. The intention was to present a Research Topic that not only enriches the academic landscape but also provides practical insights for athletes, coaches, and stakeholders involved in adapted and (dis)ability sports.

Of particular note, this Research Topic drew attention from academics and practitioners from a range of countries resulting in the publication of 14 articles improving the research knowledge of the following topics: empowerment and social inclusion through sport (five articles), performance and technical analyses in (dis)ability sports (four articles), physical and mental health in athletes with (dis)abilities (three articles), youth and mentorship in (dis)ability sports (one article), and barriers and accessibility in (dis)ability sports (two articles).

The exploration of empowerment and social inclusion through sport was explored across five studies, each highlighting different aspects of how sports participation impacts athletes with (dis)abilities. Participation in sports empowers individuals by enhancing self-confidence, providing a sense of agency, and helping athletes redefine their personal identities, as explored by Alhumaid et al. in their study on Saudi women with physical impairments. Motivation, both intrinsic, such as the drive for self-improvement, and extrinsic, like recognition from others, plays a crucial role in overcoming barriers, including structural constraints and societal stigma, as highlighted by Sarol's research on wheelchair basketball players. The psychological benefits of sports, particularly improvements in wellbeing and life satisfaction, are well-documented across the studies. Puce, Okwen, et al.'s critical review emphasizes the multidimensional nature of wellbeing, while Van Biesen and Morbee's study shows how Paralympic athletes safeguarded their mental health during the Tokyo 2020 postponement through adaptive motivational profiles. Puce, Biz, et al.'s large-scale survey further reveals that para-athletes exhibit higher levels of hedonic wellbeing compared to (dis)abled individuals not involved in competitive sports. Adapted and (dis)ability sports also act as a vital tool for social inclusion, enabling athletes to form connections, gain social recognition, and challenge feelings of exclusion, as demonstrated by Alhumaid et al.. Despite these benefits, however, many athletes still face significant obstacles in accessing sports, from resource limitations to societal attitudes, highlighting the need for continued efforts to create inclusive environments that support their full participation.

Several articles in this Research Topic focus on performance analysis and technical evaluations in adapted and (dis)ability sports, showcasing critical contributions to the field (*n* = 4). Becerra-Muñoz et al. provided an analytical insight into women's wheelchair basketball lineups at the Tokyo 2020 Paralympic Games, focusing on the impact of game-related statistics on lineup efficiency and success. This work further highlighted the importance of specific performance metrics, such as field goal efficiency and assists, in informing coaching decisions. Similarly, Suárez-Iglesias et al. examined the physiological demands of adaptive seated slalom waterskiing, comparing traditional and alternative deep-water start techniques for athletes with paraplegia, underscoring the need for tailored training programs. Meanwhile, Minder et al. investigate the neuromuscular activation and perceived exertion in wheelchair propulsion, revealing critical insights into performance fatigability and potential shoulder injury risks. While, Arnet et al. analyzed the biomechanical properties of treadmills used in exercise testing for elite wheelchair athletes, emphasizing the significance of standardized equipment validation for accurate performance assessments. Collectively, these studies contribute to a deeper understanding of the complexities of performance in adapted sports and offer valuable implications for training and competitive strategies.

Furthermore, two significant studies explored areas of physical and mental health in athletes with (dis)abilities. Urbański et al. investigated the mental health challenges faced by elite Polish athletes with disabilities during the COVID-19 pandemic, revealing that pandemic-specific coping strategies significantly predict levels of anxiety and depression. This suggests that these athletes may require tailored interventions to address the unique stressors related to the pandemic, highlighting the importance of understanding specific coping mechanisms in promoting mental wellbeing. Complementing this, Castle et al. examined the health and wellbeing of Ukrainian veterans with disabilities during a preparatory camp for the 2022 Warrior Games. Their findings indicated that while overall sleep, mood, and competition-related emotions remained relatively stable, there were notable challenges, such as low sleep duration and increased anxiety. The study reinforces the critical role of family support and the motivation to represent one's country in fostering resilience among participants. Together, these two studies shed light on the multifaceted aspects of mental health in athletes with (dis)abilities and emphasize the need for targeted support systems to enhance their overall wellbeing.

Additionally, the exploration of youth and mentorship in (dis)ability sports is a notable highlight of this Research Topic, as highlighted by Wedege et al., who examined the experiences of children with acquired brain injuries and their caregivers at peer mentorship sports camps. This longitudinal qualitative study found that Active Rehabilitation camps enriched participants' lives by fostering social connections, enhancing coping skills, and improving psychological functioning. These findings underscore the critical role of peer mentorship in promoting empowerment and social inclusion, aligning with the Research Topic's aim to highlight community support for the health and wellbeing of individuals with disabilities.

Finally, two studies surrounding barriers and accessibility in adapted and (dis)ability sports conclude the articles on this Research Topic. Meier et al. explored the challenges faced by blind and visually impaired (BVI) students in specialized physical education (PE), revealing that PE teachers can either facilitate or hinder participation. The study emphasizes the need to amplify BVI students' voices and suggests digital solutions to enhance their engagement. Carretti et al. provided a narrative review on the benefits of physical activity for balance control in visually impaired individuals, advocating for tailored exercise protocols and recognizing the crucial role of adapted physical activity specialists. Together, these studies highlight the necessity of addressing participation barriers and promoting accessible sports opportunities for individuals with disabilities.

In conclusion, this Research Topic makes a significant contribution to the field of adapted and (dis)ability sports, bringing together diverse insights from 14 articles that encompass empowerment, performance analysis, mental health, youth mentorship, and barriers to participation. However, it is important to note that the number of submissions remains modest when considering the vast network of academic and applied professionals engaged in this area, highlighting a critical challenge in knowledge sharing. National governing bodies, the International Paralympic Committee, and other organizations focused on adapted and (dis)ability sports play a vital role in fostering an environment that encourages research collaboration and the dissemination of findings. They must actively promote initiatives that inspire both researchers and practitioners to share their insights and experiences.

Notably, many of the studies presented have begun to address the fragmentation that has characterized current research, moving away from narrow, single-discipline perspectives to offer interdisciplinary insights encompassing physiological, sociological, and performance-related factors. By exploring a broader scope of adapted and (dis)ability sports and providing practical implications for athletes, coaches, and stakeholders, these articles pave the way for a more cohesive and comprehensive understanding of the field. This potential for greater collaboration and knowledge exchange is crucial for advancing the quality of research and practice in adapted and (dis)ability sports.

To build on this momentum, there is an urgent need for enhanced collaboration, resource allocation, and support for knowledge exchange initiatives. By fostering these efforts, we can create a more integrated understanding of adapted and (dis)ability sports, ultimately enriching the experiences and outcomes for athletes and stakeholders involved in this vital field.
